# Developing an Artificial Intelligence-Driven Nudge Intervention to Improve Medication Adherence: A Human-Centred Design Approach

**DOI:** 10.1007/s10916-023-02024-0

**Published:** 2023-12-08

**Authors:** Jennifer Sumner, Anjali Bundele, Hui Wen Lim, Phillip Phan, Mehul Motani, Amartya Mukhopadhyay

**Affiliations:** 1 Medical Affairs – Research Innovation & Enterprise, Alexandra Hospital, National University Health System, Singapore, Singapore; 2 Johns Hopkins Carey Business School and the Department of Medicine, Baltimore, USA; 3 Department of Electrical & Computer Engineering, National University of Singapore, Singapore, Singapore; 4 Department of Medicine, Yong Loo Lin School of Medicine, National University of Singapore, Singapore, Singapore

**Keywords:** Medication adherence, Qualitative research, Behaviour change, Design thinking

## Abstract

To improve medication adherence, we co-developed a digital, artificial intelligence (AI)-driven nudge intervention with stakeholders (patients, providers, and technologists). We used a human-centred design approach to incorporate user needs in creating an AI-driven nudge tool. We report the findings of the first stage of a multi-phase project: understanding user needs and ideating solutions. We interviewed healthcare providers (n = 10) and patients (n = 10). Providers also rated example nudge interventions in a survey. Stakeholders felt the intervention could address existing deficits in medication adherence tracking and were optimistic about the solution. Participants identified flexibility of the intervention, including mode of delivery, intervention intensity, and the ability to stratify to user ability and needs, as critical success factors. Reminder nudges and provision of healthcare worker contact were rated highly by all. Conversely, patients perceived incentive-based nudges poorly. Finally, participants suggested that user burden could be minimised by leveraging existing software (rather than creating a new App) and simplifying or automating the data entry requirements where feasible. Stakeholder interviews generated in-depth data on the perspectives and requirements for the proposed solution. The participatory approach will enable us to incorporate user needs into the design and improve the utility of the intervention. Our findings show that an AI-driven nudge tool is an acceptable and appropriate solution, assuming it is flexible to user requirements.

**Supplementary Information:**

The online version contains supplementary material available at 10.1007/s10916-023-02024-0.

## Introduction

Medication non-adherence is a persistent problem for healthcare systems, affecting 50–70% of patients with chronic disease [1, 2]. Apart from missing therapeutic gains, medication non-adherence can have significant negative clinical and economic impacts [3–6]. Accordingly, numerous efforts have gone into understanding the factors influencing medication adherence [7, 8]. The World Health Organization framework identifies five domains that impact adherence: Socioeconomic, Therapy-related, Patient-related, Condition-related, and Health system/healthcare team-related [1]. Each category can be divided into modifiable (e.g., knowledge) and unmodifiable factors (e.g., gender) [9, 10]. Despite awareness of these factors, traditional interventions have failed to account for the complexity of behaviour change and irrational choice [11, 12]. Nudges, an alternative approach, have effectively promoted healthy behaviours and improved outcomes in chronic disease [13–16].

Nudges predictably alter people’s behaviour without removing options or significantly changing costs [17]. Examples include reminders, approaches that reduce behavioural demands (e.g., home delivery of medications) or incentives to motivate action. While effective, nudges are susceptible to attention fatigue and habituation [13] but this may be overcome through digitally automating the intervention modulation [13]. Artificial Intelligence (AI) offers a promising route to adjusting interventions autonomously. The potential of AI also lies in its capacity to tailor interventions to individual's based on clinical and behavioural information [18]. In the context of medication adherence, early AI-driven nudge-based interventions have shown positive signals [15, 19], yet existing studies lack participant diversity, have limited stakeholder involvement, or used a small range of nudge interventions.

In the present study, we employed a design thinking framework to develop a digital AI-driven medication adherence nudge intervention [20]. Design thinking, an iterative human-centred approach, emphasises user needs, problem definition, idea generation, prototyping, and testing with users to create viable solutions [20, 21]. Using design thinking, we avoid well-documented technology adoption and scaling challenges that arise when stakeholders’ perspectives are overlooked [22]. This paper reports the findings from the initial phase of a multi-phase project on understanding user needs, identifying problems, and ideating solutions.

## Methods

Design thinking emphasises early iterative stakeholder involvement in intervention development rather than after an intervention has been fully developed. At the time of this report, the intervention had not been developed and we presented interviewees with a hypothetical concept. To inform the design of our intervention we collected data on i) the challenges of medication management from healthcare providers and patients; and ii) their perspectives and requirements for the proposed solution. Data were collected through interviews with healthcare providers and patients. Providers also rated example nudge interventions in a survey.

### An artificial intelligence-driven digital nudge intervention

Before the interviews, we introduced patients and healthcare providers to the concept of nudge interventions (Table 1) and how they could be used to encourage medication adherence. We presented various nudge options to explore participant’s views of the concept. We also explained that the intervention would include an AI algorithm that would deploy different nudges according to user needs and responses to individual nudges. Specific details on the type of AI technology were not discussed with the interviewees. This choice was deliberate as we recognised that AI's technical intricacies would be beyond most interviewees' ability. Instead, we objectively focused on the potential capabilities of AI rather than the mechanism behind the technology. Table 1 details different nudge categories [23] and example nudge interventions.

**Table 1:** Example nudge interventions

**Nudge category**	**Example nudge intervention**
Information is reframed	Highlights how time and money is wasted by non-adherence
Information visibility	Informs the patient of their adherence rate
Provide social reference	Access to disease support group
Change the default option	Automatic sign up to home-delivery of medication and must opt out
Change required effort	Home-delivery offered if the prescription is not collected in X days
Change options	Longer prescriptions offered
Change the consequence of an action	Collecting prescription provides the chance to win an incentive
Provide reminders	Reminder to collect or take medication
Facilitate commitment to action	Goal setting with the care team

### Development of the interview guide

We developed the patient and healthcare provider interview guides using three approaches. First, we reviewed the literature to identify common themes and issues related to the research question. We also referenced the NASSS framework (non-adoption, abandonment, scale-up, spread, and sustainability), which considers the social, organisational, and technical factors that could impact implementation [22]. The framework is common in technology development and evaluation. The project team then brainstormed to include topics not otherwise captured or that were pertinent to the local cultural and institutional contexts. For example, Singapore is a multiethnic country with four official languages (Malay, Mandarin, English and Tamil). Consideration of communication challenges and different cultural perspectives on Western medicines is needed. Finally, we allowed the topic guide to evolve naturally over time as new topics emerged from the interviews so that subsequent interviews were more productive and provided a high level of confidence that data saturation had been reached.

### Healthcare provider interviews

We recruited staff doctors and pharmacists involved in outpatient medication management at Singapore’s Alexandra Hospital between August and October 2021. Alexandra Hospital is a public general hospital offering inpatient acute care in medical and surgical disciplines and specialist outpatient services. We used a convenience sampling approach and asked interviewees to recommend other potential candidates for the project. All invited participants took part in the study.

Interviews were conducted using a semi-structured topic guide to elicit providers’ opinions on medication management experiences and the proposed solution to non-adherence. During the interview, the concept of nudge was explained to the participant verbally (Table 1). The intervention was introduced as a digital-based system, which would use AI to tailor nudge interventions to individuals. At the end of the interview healthcare providers were asked to rate example nudge interventions on effectiveness, acceptability, and feasibility to implement (Table 1).

### Patient and/or caregiver interviews

We purposively sampled patients from the Alexandra Hospital outpatient clinics between February 2022 and May 2022. Eligible patients and/or caregivers had at least one chronic condition (e.g., hypertension, diabetes) and were on one or more long-term medications for that condition. We recruited patients with different demographical profiles. Healthcare providers assisted in identifying eligible participants. A researcher then discussed the project, obtained consent, and conducted semi-structured interviews anchored around a topic guide to explore their experiences of medication management and the associated barriers and facilitators. We identified four nudge concepts from the healthcare provider interviews to discuss in the patient interviews. Three were selected as they were highly rated on their perceived effectiveness, acceptability, and feasibility by healthcare providers, and one nudge concept was selected because of mixed opinions. The nudge concepts were presented to patients through discussions and storyboards. Storyboards visually illustrated the intervention experience [24]. We asked the participants to broadly consider the strengths and limitations of the proposed solution and suggest key attributes for the intervention.

Staff interviews were conducted in English, while patient/caregiver interviews were conducted in English and Mandarin Chinese, according to patients’ preferences. Three female researchers, trained in qualitative research methodology, conducted the interviews (JS (PhD), AB (MPH), and HWL (MSc)). Chinese interviews were conducted with a native speaker (HWL). Following informed consent, the interviews were conducted in a neutral setting to facilitate open conversations. All the interviews were audio-recorded and transcribed verbatim. The Chinese audio files were translated by a native speaker and cross-checked by an independent native speaker to ensure accuracy. Interviews lasted for approximately 1-h.

### Analysis

Healthcare provider interviews assessed nudge interventions with yes or no ratings on their perceived effectiveness (clinical impact in terms of adherence), acceptability (reception by clinicians and patients), and feasibility (practical to implement). Qualitative data were collected and reported according to the COREQ checklist (Consolidated criteria for reporting qualitative research) [25]. We analysed data using a deductive approach, using the NASSS framework (non-adoption, abandonment, scale-up, spread, and sustainability) [22]. We chose the NASSS framework to organise the data and assess implementation factors. Data were categorised into four NASSS domains (1. The condition, 2. The technology, 3. The value proposition, 4. The adopter system) (Supplement 1). Data from the first domain is under review elsewhere and will not be discussed here. The fifth, sixth and seventh NASSS domains (The organisation, The wider context, and Embedding and adaptation over time, respectively) were excluded since the intervention isn’t currently in practice.

Three researchers (JS, AB, and HWL) independently coded the data in Microsoft^®^ Word, resolving disagreements through discussion. Qualitative data for healthcare providers and patients were coded separately, enabling insight into different perspectives and areas of overlap or contrast. All participants were assigned a unique ID (healthcare providers IDA-J and patients ID1-10), as detailed in the results section.

## Results

We conducted twenty interviews with healthcare providers (n = 10) and patients (n = 10). Recruitment stopped once data saturation had been reached. Healthcare providers had a mean age was 32.9 (± 5.3) years, and 60% were female; they included doctors (40%) and pharmacists or pharmacy assistants (60%). Most healthcare providers (60%) had six or more years of experience in practice; the remainder had five or less years’ of experience. The patient demographics are reported in Table 2.

**Table 2:** Patient interviewee characteristics

	**N = 10**
Age, years, mean (SD)	73.6 (9.3)
Female, n (%)	3 (30)
Ethnicity, n (%)	
*Chinese*	8 (80)
*Malay*	2 (20)
Educational status, n (%)	
*No formal/primary level education*	3 (30)
*Secondary*	3 (30)
*Diploma and above*	4 (40)
Living alone, n (%)	1 (10)
Chronic conditions, n (%)	
*High cholesterol*	7 (70)
*Diabetes*	7 (70)
*Hypertension*	6 (60)
*Heart disease or Stroke*	2 (20)
*CKD*	2 (20)
*Dementia*	1 (10)

*SD* Standard deviation

### NASSS domain 2: The technology

**Fig. 1:**
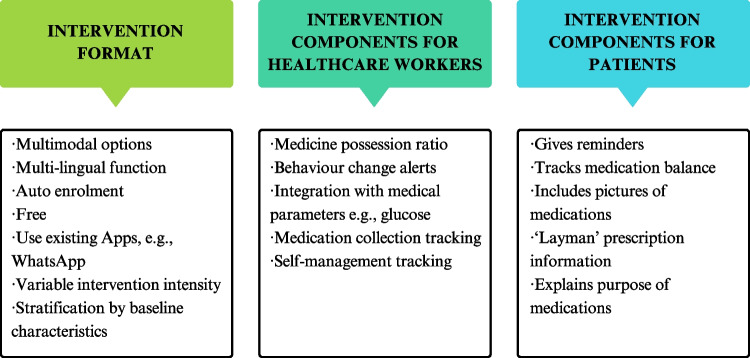
A summary of suggested intervention features

The participants identified many desired features for the proposed intervention (Fig. 1). Healthcare provider discussions often focussed on the flexibility of the system and the ability to identify specific user attributes. For instance, healthcare providers felt that elderly patients preferred the personal touch of a call, while working-age adults preferred the convenience of an app.

*IDE “Middle-aged generations are more tech-savvy and also prefer more convenience.” [app or phone call based]*

*IDH “An app is plus and minus because a human is personal touch after all, an app can never replace that.”*

Patients mirrored these comments, noting issues with technology literacy and a desire for personal interactions, which would require the proposed intervention to take a multi-modal approach.

*ID02 “A phone call may be considered old fashioned to some people. But it is the* people’s *touch.”*

Flexibility was also considered important in relation to intervention frequency. Varying the intensity of the intervention could be one way to prevent users from becoming overwhelmed or losing interest (i.e., habituation to behavioral norms or attention fatigue).

*ID02 “You know messages. We get a dozen messages everyday and we just delete it or ignore it.”*

Healthcare providers suggested stratification based on the complexity of the case and the degree of adherence. Stratifying would inform the system on the intervention type and intensity, making it personalised and more effective.

Both groups of participants reflected on the challenge of medication routines, understanding the purpose of each medication, and following instructions. Patients and pharmacists suggested simplified prescription instructions, which could include visuals of the medications, and explanations of each medication’s purpose.

*IDI “It would be even more helpful that these prescriptions are actually displayed to the patient in layman language right. Cause sometimes, I think right now the prescriptions don’t really show in layman language as well.”*

Integration with the existing systems was another point raised by interviewees. For instance, using familiar applications like WhatsApp (a common messaging app) was suggested to avoid introducing a new system. Patients also mentioned the burden of installing new healthcare apps. Hence, avoiding new app or software installation was preferred.

*ID05 “There are many apps that were launched by the government, many apps. For us who don’t have much education, we can’t understand…I can’t use.”*

At the system level, integration with existing medical databases was perceived as essential for an effective intervention. Participants emphasised the need for real-time accurate data for adherence monitoring. Limited access to complete prescription records was acknowledged, but interviewees were optimistic this would resolve with the new electronic medical record system.

*ID A “Hard for the AI. But if you are moving towards EPIC [a harmonized electronic medical record system] then it’s fine for checking for adherence.”*

Other considerations for the intervention include data entry, data accuracy and complexity.

*ID A “It would be challenging to ascertain reliability.” [patient inputting data] If it’s a caregiver I think they answer truthfully [re data entry].*

*ID C “So some patients maybe do not want to expose too much of their confidential information to the system.”*

### NASSS domain 3: Value proposition

Most expressed an interest in the solution and felt that it would enhance existing processes, particularly screening for non-adherers.

*IDE “How do I keep track, or how do I monitor patients that do not collect their medicine? We do not have that now, we don’t monitor each patient that we have.”*

Clinicians reported the need for such a solution due to existing support gaps but emphasised the importance of context. For example, older age was often reported as a barrier due to technological literacy or low interest, yet some older adults are curious about trying new things if supported. Others noted that caregivers would find the intervention appealing even if the patient did not.

*IDG “50-60% of them are quite tech savvy [patients attending now]….If not, they would have family members who are very interested to make use of this.”*

However, patient’s opinions were divided on this point. Some reported that technological literacy was prohibitive to this type of intervention as well as phone ownership.

*ID02 “If you don’t own a phone how are you going to get the messages? Old folks do not have smart phones”*

*ID05 I really don’t know how to use, I don’t know anything related to digitalization. I don’t even really know how to use a phone”*

**Fig. 2:**
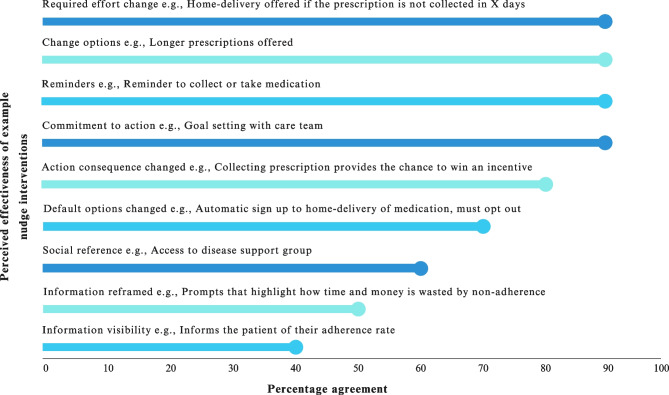
Perceived healthcare provider agreement on nudge interventions effectiveness

**Fig. 3:**
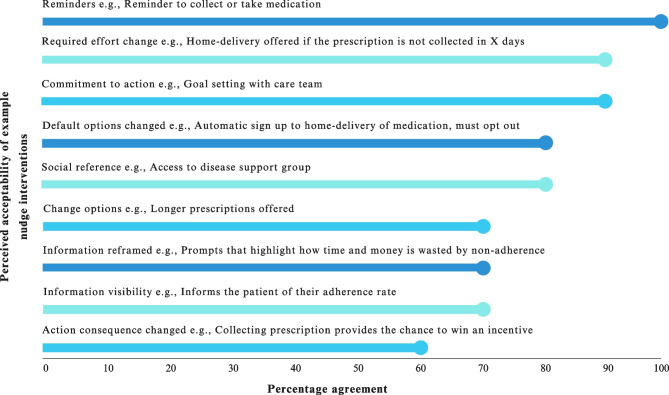
Perceived healthcare provider agreement on nudge interventions acceptability

**Fig. 4:**
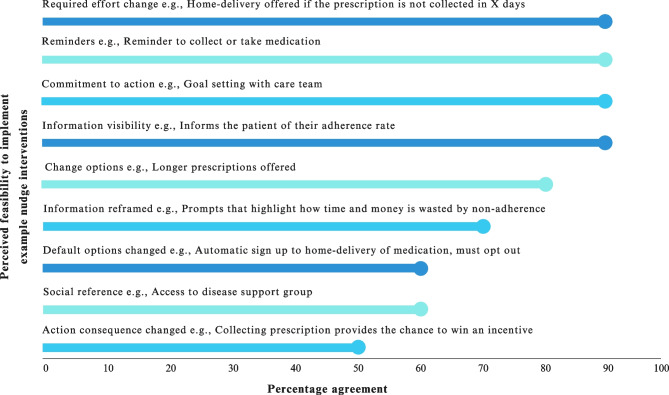
Perceived healthcare provider agreement on nudge interventions feasibility to implement

During the interviews, healthcare providers rated example nudge interventions in terms of their perceived effectiveness, feasibility to implement, and acceptability to users (Figs. 2, 3, and 4).

Patients were interviewed on their perspectives of four nudge interventions (Table 3). Patients felt a need for reminders and remote contact from healthcare providers. While healthcare providers rated home delivery highly, patients felt it was not for them. Participants reported that their preference for self-collection was rooted in a desire to see the doctor in person, exercise, and check that the medication dispensed was correct. Home delivery was deemed more suitable for those with mobility issues. Healthcare providers reported that incentives would effectively encourage medication adherence, while patients were particularly critical of this idea. Patients believed they were responsible for managing their medication, and incentives were the wrong motivation. Participants were also concerned that this might be mistaken for a scam. Finally, participants emphasised the importance of keeping caregivers involved, regardless of the intervention.

**Table 3:** Participants reflections on example nudge interventions

**Nudge intervention**	**Example quotes**
**Reminders to encourage adherence.**	*IDE “Like we have very busy, tight schedule at work, they might have overlooked th*e*ir medication collection or running out of medications. So with this prompt it could help them”* *IDJ “So the main challenges we have is our elderly who live alone… they tend to forget their medications. And currently there’s, there’s no real service in place to serve as the daily reminder.”* *ID07 So there must be a system for you to manage you don’t think too much it becomes automatic [medication management]*
**Home-Delivery offered if medication not collected.**	*ID07 “Because I like to see the doctor and ask the doctor questions, unless my case is very stable ….then I don’t mind”* *ID08 “Firstly it is because I’m an active person, I like to exercise. Secondly, I want to know the person giving the medicine. Whether right or wrong. You can see. And say it on the spot”* *ID10 “People can do other things rather than just deliver. Because I can do that myself for the moment. If one day I cannot walk or maybe I just need the help, I think this is good.”*
**Healthcare provider contact, if medication not collected.**	*ID07 “This is perfect, this is what should be done. You got to basically re-educate, I think this is very good system.”* *ID10 “I think it helps. Because sometimes some issue with medicine if not we don’t know what is happening, why should I take this medicine.”* *ID05 “You mentioned that they will call him, if he is not staying with me…his appointments these kind all will send to my phone, then I also don’t know if he takes the medications.”*
**Incentives to motivate adherence.**	*ID02 “I think it’s helpful because in the current situation I think people will always appreciate a little bit extra incentive.”* *ID05 “There are too many scammers on the phone recently, I will take it as possible phishing scam.”* *ID 07 “It’s like treating the kid to do something, in principal it’s not right but you got to give something so he does it. I mean collecting medicine is a responsibility, getting your medicine is for your own benefit.”*

### NASSS domain 4: The adopter system

Role changes were anticipated by clinicians, patients, and caregivers if this system was implemented. For example, patients would register on the system while healthcare providers would monitor results. However, concerns over ownership of the system were noted. It was agreed that the pharmacy department should handle medication management matters, but limitations like data access, time, and resources were reported. Clinicians must also be prepared to use traditional methods, like phone calls, to engage less tech-savvy individuals.

For patients, a willingness to share data and interact with the system is important. Using the intervention must be supported by appropriate training and the availability of messages in different languages. The intervention must also avoid creating an additional burden, which may lead to poor engagement. Finally, access to technology should not be assumed, and engagement of the caregiver rather than the patient may be more appropriate in certain cases.

## Discussion

Nudge interventions have gained traction in healthcare, and early research has shown promise in improving medication management [15, 19]. To overcome the limitations of existing studies, we used a design-thinking approach to co-develop a new nudge behaviour change intervention. Interviews identified the need for better medication tracking and enhanced patient support. Participants discussed user preferences, including the intervention's essence and structure, customisation possibilities, and system requirements. Healthcare providers and patients proposed a multi-modal approach to address technology literacy and ensure personalised interactions. They also advised leveraging existing applications or software where possible, varying the intervention intensity to reduce burden, and simplifying or automating the data entry requirements. Reminders and remote contact from healthcare providers were identified as the two most effective interventions. By incorporating these insights, we can create a patient-centric intervention.

Health technologies can enhance patient care, yet often face resistance and underutilisation when implemented [26]. Lack of user involvement in technology development is one explanation for poor uptake. Usability, usefulness, cost, and personal security concerns are often overlooked when end-users are not part of concept development [26]. Participatory methods, like design thinking, have emerged to identify and incorporate consumer perspectives into product development [20, 27, 28]. Design thinking facilitates the recognition of real user issues, fosters collaboration among diverse stakeholders, and ensures practical solutions by prioritising users in the design process [20, 27, 28]. Early understanding of stakeholder needs and expectations can prevent costly and time-consuming modifications later in development. Furthermore, early stakeholder engagement establishes buy-in and ensures the intervention is relevant, effective, and sustainable [29].

Initial studies using nudges for medication adherence have shown encouraging effects, but efficacy has yet to be established [15, 19]. Besides trial evidence, considering the effects and feasibility of nudges in real-world settings is crucial. Exploring the optimal implementation of nudges, who they work best for, and their long-term viability requires additional research. Nudges in other domains like physical activity, and smoking cessation, appear effective, but their benefits can be impacted by contextual factors [30–32]. In medication adherence, one trial found data access and the ability to identify medication refill gaps were significant feasibility challenges [15]. Our participants also raised this issue, noting concerns about the available data's completeness, timeliness and accuracy. Ongoing digitisation efforts, locally and internationally should address such issues [33, 34], but recognising these domains underscores the importance of understanding how interventions function in real-world scenarios.

Beyond data logistics, developers must consider ethics, data privacy and regulatory requirements in AI applications in healthcare. The growing interest in AI within healthcare has motivated the formulation of AI principles, laws and checklists by professional bodies, endorsed by organisations such as the World Health Organization and the American Medical Informatics Association, and the European Commission [35–39]. These guidelines cover the various facets of AI development and use, emphasising the importance of ethical, user-centred, and responsible innovation within the healthcare domain [35–39]. The aim is to ensure AI systems are technically robust, ethically sound, user-centred, and responsibly developed to derive positive societal impacts.

In the next phase of our project, we will co-develop message scripts that will be used to communicate nudges to patients. The co-design approach will ensure that the messaging content is understood and is appropriate from a patient and provider’s perspective. We will then create a prototype system and invite target users to assess the system’s usability, feasibility, and acceptability in a real-world scenario. The pilot evaluation will also help us understand the implementation barriers and enablers, which may impact future scalability and sustainability.

### Strengths and limitations

Stakeholder interviews allowed for the collection of rich data on the current medication management landscape and perspectives of the proposed solution. This data will help us to develop a new intervention informed by stakeholders. However, we only collected data from a single institution, from limited professional groups (physicians and pharmacy staff) and patients with chronic disease; there may be other perspectives we did not capture, for example, care managers or younger adults with chronic disease. We aim to overcome this in future studies by involving a greater breath of participants. Finally, these results may not be generalisable to other countries, with their own contextual considerations. However, through this publication, we hope to inform others of the issues they should consider in their product or service developments.

## Conclusions

While early studies on AI-driven nudge-based interventions are encouraging, their development often lacked stakeholder involvement, potentially hindering acceptability and scalability. Employing a design thinking approach, we addressed such limitations by actively engaging stakeholders, identifying user requirements and anticipating potential challenges with our nudge-based intervention. We found that creating a flexible system that accommodates user abilities and preferences is crucial in developing a patient-centric intervention that meets the needs of all stakeholders. Other suggestions include leveraging existing applications, varying intervention intensity to reduce fatigue and maintain engagement, and simplifying or automating data entry requirements where feasible. Our future research will focus on developing and testing a prototype in real-world settings.

## Supplementary Information

Below is the link to the electronic supplementary material.

Supplementary file1 (DOCX 18 KB)

Supplementary file2 (PDF 416 KB)

## Data Availability

Data is available upon reasonable request.
