# Supplementary material for: Developing an Artificial Intelligence-Driven Nudge Intervention to Improve Medication Adherence: A Human-Centred Design Approach
Source: J Med Syst. 2023 Dec 8;48(1):3. doi: 10.1007/s10916-023-02024-0 (PMC10709244; doi:10.1007/s10916-023-02024-0)
Supplement: Supplementary file 1 — Supplementary file1 (DOCX 18 KB) [file 10916_2023_2024_MOESM1_ESM.docx]

**Supplement 1: NASSS domains 1-4, classifications, and rationale**

| **Domain/Questions** | **Classification** | **Rationale** |
| --- | --- | --- |
| **Domain 1: The condition or illness**  1A. What is the nature of the condition  or illness? | Simple | Chronic diseases are generally well-characterised requiring long-term therapeutic management. |
| 1B. What are the relevant sociocultural factors and comorbidities? | Complicated | Medication adherence (in the context of chronic disease) may be impacted by multiple factors, including sociocultural factors and the presence of comorbidities. The WHO medication non-adherence framework identifies 5 groups of factors linked to medication adherence. |
| **Domain 2: The technology**  2A. What are the key features of the  technology? | Complicated | The intervention will leverage artificial intelligence to learn what works best for whom and modulate interventions accordingly. The system will require training and optimisation on what data capture is needed. |
| 2B. What kind of knowledge does  the technology bring into play? | Simple | The technology would enable monitoring of medication, something which is not routinely or consistently tracked by staff or patients currently. |
| 2C. What knowledge and/or support  is required to use the technology? | N/A | Technology not yet developed. |
| 2D. What is the technology supply  model? | N/A | Technology not yet developed. |
| **Domain 3. The value proposition**  3A What is the developer’s business case for the technology (supply-side  value)? | N/A | Technology not yet developed. |
| 3B What is its desirability, efficacy, safety, and cost-effectiveness  (demand-side value)? | Simple | The technology is desirable to stakeholders and perceived as being a viable and effective solution. Cost can be minimised through autonomous AI technology, which can modulate the intervention without the need for provider input. |
| **Domain 4. The adopter system**  4A What changes in staff roles, practices, and identities are implied? | Complicated | Staff will have to take on new roles, such as monitoring the patients using the system. This will require new workflows and responsibilities, which will take time to develop. |
| 4B What is expected of the patient (and/or immediate caregiver)—and is this achievable by, and acceptable to them? | Simple | Routine tasks would be expected, such as responding to messages. Participants perceived this as feasible so long as efforts are made to simplify or automate data collection were possible. |
| 4C What is assumed about the extended network of lay caregivers? | Complicated | For some, lay caregivers handle matters relating to medication collection and management. In these cases, the lay caregiver would be expected to interact with the system. |
